# Determinants of the perceived financial threat of COVID-19 and implications for household economic stability: an application of the partial proportional odds model

**DOI:** 10.1186/s13561-025-00637-4

**Published:** 2025-08-22

**Authors:** Maru Zewdu Kassie, Seyifemickael Amare Yilema, Alebachew Taye Belay, Najmeh Nakhaei Rad, Ding-Geng Chen

**Affiliations:** 1 Department of Statistics, College of Natural and Computational Sciences, Assosa University, Assosa, Ethiopia; 2 Department of Statistics, College of Natural and Computational Sciences, Debre Tabor University, Debre Tabor, Ethiopia; 3 Department of Statistics, University of Pretoria, Pretoria, South Africa; 4 College of Health Solution, Arizona State University, Phoenix, 85004 USA

**Keywords:** COVID-19, Household, Perceived financial threat, Partial proportional odds model, Economic stability

## Abstract

**Background:**

The COVID-19 pandemic has been one of the most significant global health crises in recent years. This study aimed to assess the determinants of perceived financial threat of COVID-19 and its implications on household’s economic stability in Ethiopia.

**Methods:**

A cross-sectional study was conducted on 3058 households from the third round of the COVID-19 high-frequency phone survey of households (HFPS-HH) data, executed by the Central Statistical Agency of Ethiopia in partnership with the World Bank. The data were extracted and managed using STATA version 17. A partial proportional odds model was applied to assess the significant predictors that affect the perceived financial threat of COVID-19.

**Result:**

The analysis revealed that 79.9% of respondents (95% CI: 78.5–81.4%) experienced some level of financial threat from the COVID-19 pandemic, with the majority of them (61.7%; 95% CI: 60.0–63.4%) perceiving it as a substantial financial threat. Key factors of financial threat included Age [AOR = 1.280, *P* = 0.008]; COVID-19 illness worry for substantial threat (AOR = 0.546, *p* < 0.001), for moderate threat (AOR = 0.562, *P* = 0.005)]; Ability to buy medicine for substantial threat [AOR = 0.546, *p* < 0.001], for moderate threat [AOR = 0.562, *p* = 0.005]; employed [AOR = 1.310, *p* = 0.014]; engagement in additional income activities for substantial threat [AOR = 3.428, *p* < 0.001], and for moderate threat [AOR = 3.043, *P* = 0.018].

**Conclusion:**

The findings revealed that a significant proportion of respondents perceived COVID-19 as a financial threat, which adversely affected their economic stability. Vulnerability to financial threat was notably higher among older individuals, the unemployed, those unable to afford essential medicine, and respondents who expressed heightened concern about illness. In contrast, engagement in additional income-generating activities served as a protective factor. These results underscore the need for policymakers to prioritize inclusive social protection systems, expand access to affordable healthcare, promote employment opportunities, and facilitate income diversification. Such interventions are critical to enhancing household economic resilience and enabling a rapid response to future public health and economic crises. Additionally, future research should consider longitudinal designs to track changes in perceptions over time and incorporate broader economic indicators.

## Introduction

The COVID-19 pandemic has been one of the most significant global health crises in recent years (Pollard et al. 2020). As of the end of 2022, the world had recorded over 650 million confirmed cases and more than 6.6 million deaths, making it one of the deadliest pandemics in modern history (Organization 2022). The burden was not distributed equally across regions. Africa reported over 11.5 million cases and around 252,378 deaths, with many experts warning that low testing capacity likely led to significant underreporting (CDC 2020). Within the continent, sub-Saharan Africa faced compounded challenges due to under-resourced health systems, widespread poverty, and high rates of informal employment, which limited the effectiveness of lockdowns and economic recovery efforts (Aga and Maemir 2021; Hill and Narayan 2020). By the end of 2022, Ethiopia had reported over 500,000 COVID-19 cases and 7,500 deaths. Beyond the health impact, the pandemic caused severe economic disruption due to government measures like mobility restrictions, business closures, and supply chain issues, which heavily affected household incomes, particularly in urban areas. Vulnerable groups, such as those in informal employment and rural communities reliant on remittances and agriculture, were especially at risk (WHO), 2023; Wieser et al. 2020a).

From an economic perspective, the COVID-19 pandemic significantly disrupted the livelihoods of individuals globally, causing both social and economic crises (Khan et al. 2021; Maital and Barzani 2020; Pollard et al. 2020). Even though COVID-19’s immediate health effects have been extensively researched, its long-term economic impacts especially, in low and middle-income countries like Ethiopia remain a crucial topic of research (Janssens et al. 2021; Josephson et al. 2021; Mulugeta et al. 2021).

The pandemic affected millions of livelihoods in Ethiopia through lockdowns, travel restrictions, and business closures, implemented to stop the spread of the virus (Goshu et al. 2020; Kassie et al. 2023; Mulugeta et al. 2021). Most Ethiopian households, especially those in rural areas, depend on agriculture and informal economic activity, both of which are extremely susceptible to outside shocks. In contrast, urban households had to deal with declining earnings, job losses, and rising living expenses (Goshu et al. 2020; Mamo et al. 2022; Mulugeta et al. 2021). The pandemic has significantly impacted households’ psychological and financial well-being, leading to reduced spending, higher debt, and unhealthy coping strategies (Elshaer et al. 2022; Syed et al. 2021; Vieira et al. 2021).

Previous studies (Ababulgu Abasimel and Wana Fufa 2021; Fetzer et al. 2021; van Der Velden et al. 2020) revealed that individuals with lower income and unstable employment status were highly threatened by COVID-19 on their economic stability. Another study on the economic and financial influences of COVID-19 shows its impact on markets and institutions, identifies important areas for future research, and emphasizes lessons from previous pandemics and the major challenges caused by COVID-19 (Goodell 2020). The perceived financial threat of COVID-19 is acute in Ethiopia, where the poverty rate is high and access to social safety nets is limited (Ababulgu Abasimel and Wana Fufa 2021; Demiessie 2020). To inform future policy interventions and foster resilience against similar crises, it is important to understand how households perceive the financial threat posed by the pandemic and how these views affect their economic stability.

There is a dearth of information describing the relationship between financial threat perception and the economic outcomes of COVID-19 in Ethiopia; even the available studies have mainly focused on disease prevalence and immediate impacts. Most of the previous studies about the perceived financial threat of COVID-19 were qualitative research and lacked robust statistical modeling. To fill this gap, this study utilized an advanced partial log odds model (generalized ordered logistic regression model) to analyze the determinants of the perceived financial threat of COVID-19 and its implications on the household’s economic stability. As the proportional odds assumption was tested and violated, the authors utilized this robust model (Fullerton and Xu 2012; Peterson and Harrell Jr 1990). Therefore, the main objective of this study is to determine and assess the perceived financial threat of COVID-19, and its implications for the household’s economic stability.

## Methods and materials

### Study design

This study employed a cross-sectional study design to assess the determinants of perceived financial threat of COVID-19 and its implications for household’s economic stability in Ethiopia. Although the COVID-19 High-Frequency Phone Survey of Households (HFPS-HH) was originally designed as a longitudinal panel survey tracking the same respondents across multiple rounds, the current analysis focused exclusively on data collected during the third round.

### Source of data and data collection procedures

The data used in this study were obtained from the third round of the HFPS-HH survey, implemented by the Central Statistical Agency (CSA) in collaboration with the World Bank (Wieser et al. 2020b). The survey covered both rural and urban households across all regions of Ethiopia, ensuring broad geographic representation. Given that the survey was conducted via mobile phone, respondents were selected from households with accessible and registered phone numbers, which may slightly favor urban or more connected rural households. Nevertheless, the sampling frame was nationally representative, and weighting adjustments were applied to mitigate bias related to phone ownership and accessibility. The respondents of the survey were adult members of the household, most commonly the household head or another informed member capable of answering economic and demographic questions. The age of respondents ranged from 18 years and above.

The original sample consisted of 3,249 households, of which 3,058 responded to the round 3 (R3) calls. The 15-minute questionnaire covered various topics including access to basic needs, children’s educational activities during school closures, employment dynamics, household income and livelihood, income loss and coping strategies, food security, and assistance received. The dataset includes important socio-economic and demographic variables, including the perceived financial threat of COVID-19. In addition to subject-level variables, the data incorporates geographical identifiers such as region, enumeration area, zone, woreda, and kebele. These were considered for multilevel modeling due to their hierarchical structure. However, the unequal distribution of respondents across clusters, with each cluster containing a small number of observations, rendered these geographical variables unsuitable for multilevel analysis.

### Variables under study

The primary outcome variable for this study was the Perceived Financial Threat of COVID-19, categorized into four ordered levels:

$$\begin{gathered} Perceived\:Financial\:Threat\:of\:COVID - 19\: = \\ - \:\left\{ {\begin{array}{*{20}{c}}{0,\:\:Not\:a\:threat\:at\:all\:} \\ {\:1,\:\:\:\:Not\:much\:of\:a\:threat} \\ {\:2,\:\:A\:\bmod erate\:threat} \\ {\:3,\:\:A\:subs\tan tial\:threat} \end{array}} \right. \\ \end{gathered} \ $$

Independent variables included age of the respondent (measured in years), gender of the household head (male, female), gender of the respondent (male, female), respondent relation to household head (head, spouse, son/daughter, others), residence (rural, urban), covid-19 illness worry (very worried, somewhat worried, no too worried, no worried at all), able to buy enough medicine (no, yes), able to buy enough teff/injera (no, yes), employment status (unemployed, employed), engaged in additional income activities (no, yes), relied on savings (no, yes), and household lacked nutritious food (no, yes).

The unit of analysis for this study is the household. One adult respondent was interviewed per household, and their relation to the household head (e.g., head, spouse, child, other) was recorded. Variables related to both the respondent (e.g., gender, relation to head) and the household head (e.g., gender) were included to capture potential intra-household variations in perceived financial threat.

Key independent variables—such as the ability to afford essential items (e.g., medicine and teff/injera), reliance on household savings, and engagement in additional income-generating activities—are closely linked to economic stability. These factors not only influence the level of financial threat perceived by households but also serve as indicators of their economic resilience during crises. For instance, household engagement in supplementary income-generating activities served as a buffer against economic instability. Understanding these relationships is crucial for informing policy interventions aimed at strengthening household economic resilience during public health emergencies and future economic shocks.

## Methods of data analysis

### Descriptive data analysis

It is a descriptive-analytical tool that is used to analyze the quantitative data by use of cross-tabulation. Cross tabulation establishes the count of the respondents in each category of dependent variable across all categories of independent variables by use of a two (more) dimensional table. Moreover, it presents full information about the relationship between variables. This analysis technique is used in our study to know the percentage of households in each levels of perception of financial threat, and also to reveal the association between each predictor variable with the perceived financial threat of COVID-19.

### Partial proportional odds model analysis

Logistic regression is a model used in regression analysis to model the relationship between a categorical response variable and any type of independent variables. The dependent variable in this model is ordinal (having more than two ordered categories). Since the perceived financial threat has four ordered categories (a substantial threat, a moderate threat, not much of a threat, and not a threat at all), the ordinal logistic regression model is an appropriate modeling approach. However, upon testing, the proportional odds assumption was violated for some predictors. Consequently, this study used the robust model, a partial proportional odds model (PPOM) to analyze the determinants of the perceived financial threat of COVID-19, and its impact on the household’s economic well-being (Fullerton and Xu 2012; Peterson and Harrell Jr 1990). In this study, the perceived financial threat of COVID-19 was initially categorized into four groups: not a threat at all, not much of a threat, a moderate threat, and a substantial threat. However, because the frequency of the ‘Not much of a threat’ category was small and its concept closely related to ‘not a threat at all,’ we decided to combine both ‘not much of a threat’ and ‘not a threat at all,’ renaming the category ‘a small threat’ for the final analysis, which includes three ordinal categories.

### Model specification

Let Y denote the dependent variable, “Perceived Financial Threat of COVID-19,” with three ordered categories (a small threat, a moderate threat, and a substantial threat). The PPOM is specified as:

$$\:logit\:\left[p\left(Y\le\:j|X\right)\right]=\:{a}_{j}+{\beta\:}_{j}^{{\prime\:}}X,\:\:\:J=1,\:2,\:3$$

Where:

$\:p\left(Y\le\:j|X\right)$ is the cumulative probability of the outcome being in category $\:j$ or lower.

$\:j$ represents the thresholds (1 for “a small threat”, 2 for “a moderate threat”, and 3 for “a substantial threat”).

$\:{\alpha\:}_{j}$ is the intercept for threshold $\:j$

$\:{\beta\:}_{j}$ is the vector of regression coefficients for the factors at threshold $\:j$.

$\:X$ is the vector of explanatory variables.

This model relaxes the proportional odds assumption by allowing $\:{\beta\:}_{j}$ to vary across thresholds for factors where the assumption doesn’t hold. For factors that satisfy the proportional odds assumption, the same $\:\beta\:$ is used across all thresholds.

The model was estimated using the **gologit2** package in STATA, the statistical software that supports the estimation of PPOM. This package also facilitated the testing of the proportional odds assumption for each predictor, allowing the model to appropriately account for non-proportional relationships.

The results were presented as adjusted odd ratios (AORs) with corresponding 95% confidence intervals. The model outputs included threshold-specific regression coefficients, indicating how each factor influenced the cumulative probabilities of being in higher versus lower categories of the dependent variable. For variables that satisfied the proportional odds assumption, a single coefficient was reported. For those violated the assumption, threshold-specific coefficients provided insights into the varying effects across the outcome categories.

### Model diagnostics in the partial proportional odds model

To verify the proportional odds assumption, a standard ordinal logistic regression model was first fitted. The Brant test was then applied to assess whether each independent variable met this assumption. This test evaluates whether the relationship between each independent variable and the log-odds of the outcome remains constant across the ordered categories of the dependent variable. A significant p-value (*p* < 0.05) for either the overall model or an individual factor indicates a violation of the assumption. In such cases, PPOM is appropriate, as it accommodates variables that do not meet the assumption by allowing their effects to vary across outcome thresholds (Agga and Scott 2015; Williams 2006).

## Results

### Description of the perceived financial threat of COVID-19

**Fig. 1:**
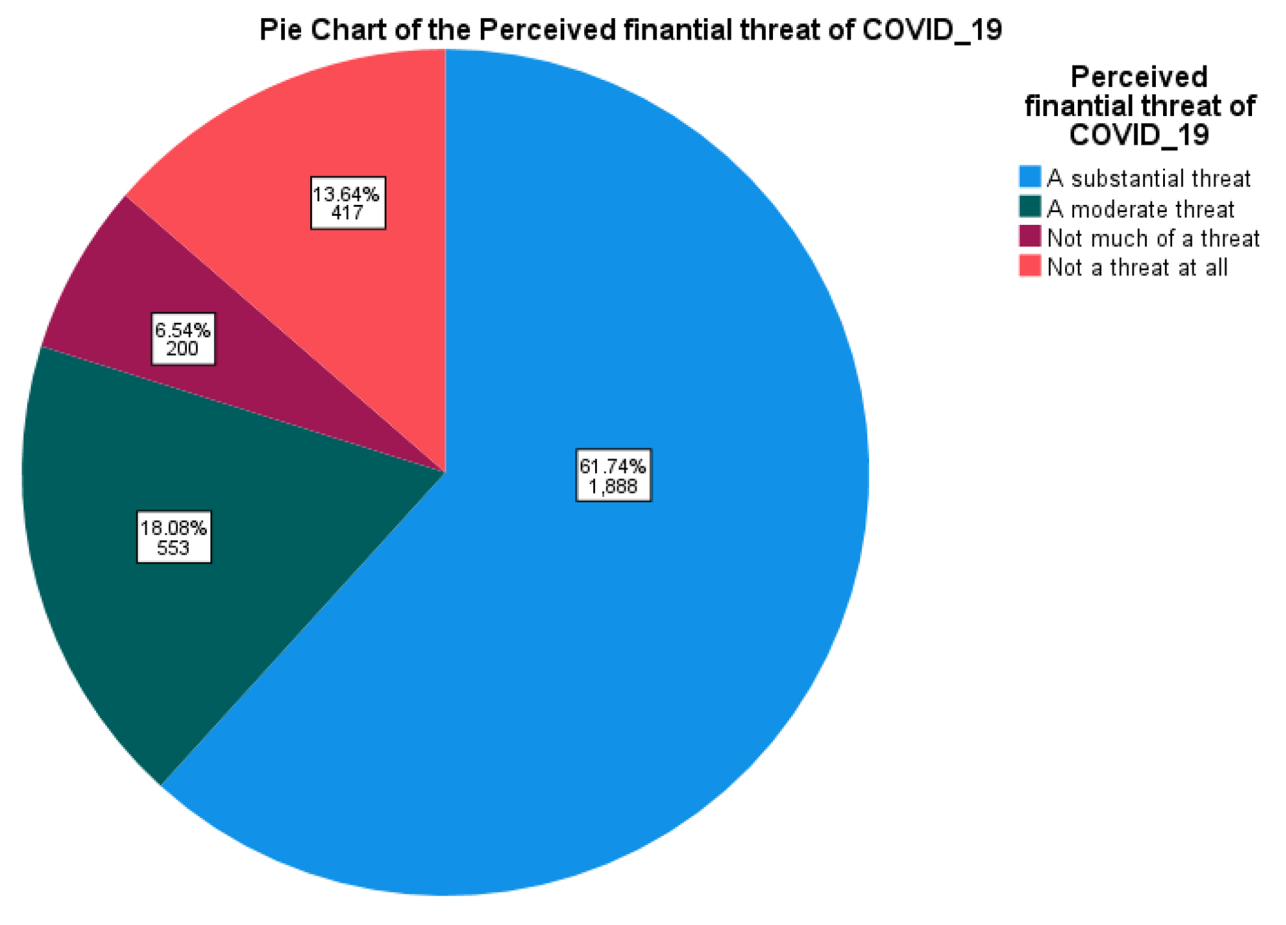
Pie chart of the perceived financial threat of COVID-19

From a total of 3,058 respondents who were interviewed in the third round of the HFPS-HH survey, the majority, 1,888 (61.7%), reported COVID-19 as a substantial financial threat. Additionally, 553 (18.1%) perceived COVID-19 as a moderate threat, 200 (6.5%) reported it as not much of a threat, and 417 (13.6%) stated that COVID-19 posed no financial threat at all to their livelihoods. The results revealed that 79.9% of respondents (95% CI: 78.5–81.4%) experienced some level of financial threat from the COVID-19 pandemic, with the majority of them perceiving it as a substantial financial threat Fig. 1.

### The perceived financial threat across their socio-economic and demographic characteristics

The cross-tabulation analysis presented in Table 1 shows significant associations between the perceived financial threat of COVID-19 and a set of independent variables. The gender of the household head plays an important role; a higher number of male-headed households (42%) reported COVID-19 as a substantial financial threat compared to female-headed households (19.8%). This relationship was statistically significant, with a chi-square p-value of 0.017, indicating a significant gender difference in economic vulnerability. However, the gender of the respondent was found to be insignificant (p-value = 0.382), suggesting that the gender of the individual respondent may not independently determine the perceived financial thereat of COVID-19.

**Table 1:** The perceived financial threat across their socio-economic and demographic characteristics

Variables	Category	Perceived financial threat of COVID-19				X^2^ *p*-value
		Substantial threat	Moderate threat	No much threat	No threat at all	
		Count (%)	Count(%)	Count (%)	Count(%)	
Gender of HHH	Male	1283(42)	409(13.4)	149(4.9)	283(9.3)	0.017
	Female	605(19.8)	144(4.7)	51(1.7)	134(4.4)	
Gender of the respondent	Male	1169(38.2)	362(11.8)	125(4.1)	252(8.2)	0.382
	Female	718(23.5)	191(6.2)	75(2.5)	165(5.4)	
Respondent relation to HHH	Head	1569(51.3)	438(14.3)	162(5.3)	337(11.0)	0.670
	Spouse	167(5.5)	57(1.9)	20(0.7)	42(1.4)	
	Son/Daughter	123(4.0)	46(1.5)	13(0.4)	29(0.9)	
	Others	28(0.9)	12(0.4)	5(0.2)	9(0.3)	
COVID-19 illness worry	No worried at all	200(6.5)	74(2.4)	33(1.1)	209(6.8)	0.000
	No too worried	134(4.4)	82(2.7)	47(1.5)	49(1.6)	
	Somewhat worried	286(9.4)	172(5.6)	46(1.5)	63(2.1)	
	Very worried	1268(41.5)	225(7.4)	74(2.4)	96(3.1)	
Able to buy enough medicine	No	53(8.9)	4(0.7)	2(0.3)	1(0.2)	0.000
	Yes	322(54.0)	99(16.6)	35(5.9)	80(13.4)	
Able to buy enough teff/injera	No	221(16.1)	32(2.3)	15(1.1)	14(1.0)	0.000
	Yes	672(49.0)	196(14.3)	60(4.4)	161(11.7)	
Employment status	Unemployed	464(15.2)	111(3.6)	36(1.2)	85(2.8)	0.021
	Employed	1424(46.6)	442(14.5)	164(5.4)	332(10.9)	
Additional income activities	No	972(77.8)	158(12.7)	32(2.6)	58(4.6)	0.003
	Yes	15(1.2)	8(0.6)	2(0.2)	4(0.3)	
Relied on savings	No	657(52.6)	136(10.9)	28(2.2)	47(3.8)	0.000
	Yes	330(26.4)	30(2.4)	6(0.5)	15(1.2)	
Household lacked nutritious food	No	928(30.4)	347(11.3)	137(4.5)	310(10.1)	0.000
	Yes	960(31.4)	206(6.7)	63(2.1)	107(3.5)	
Residence	Rural	538(17.6)	212(6.9)	66(2.2)	118(3.9)	0.000
	Urban	1350(44.1)	341(11.2)	134(4.4)	299(9.8)	

The respondent’s relation to the household head showed that heads of households (HHH) perceived COVID-19 as a substantial financial threat more frequently (51.3%) than spouses or children. However, this association was not statistically significant (*p* = 0.670). Additionally, the degree of worry about family or self-falling ill with COVID-19 demonstrated a very strong relationship with perceived financial threat (*p* < 0.001). Respondents who were “very worried” overwhelmingly perceived the pandemic as a substantial financial threat (41.5%), highlighting the direct impact of health concerns on economic perceptions.

Financial insecurity emerged as a critical determinant. Respondents who were able to buy enough medicine (54.0%, *p* < 0.001) or teff/injera (49.0%, *p* < 0.001) were significantly more likely to perceive COVID-19 as a substantial threat. These findings suggest that, even if the respondents were in a stable economic situation, they perceived COVID-19 as a financial threat, as the pandemic affects all individuals regardless of their economic status. This significant difference may have occurred because the majority of respondents stated that, even if they were not receiving aid, they could still secure their basic needs such as food, medication, etc. Employment status also played a significant role: 46.6% of employed individuals reported COVID-19 as a substantial financial threat, compared to only 15.2% of unemployed individuals. This suggests that employed individuals may have greater financial responsibilities compared to unemployed individuals.

Additionally, financial coping mechanisms, such as engaging in additional income-generating activities or relying on savings, were important factors of the perceived financial threat of COVID-19. Households not engaged in additional activities were more likely to report a substantial threat, accounting for 77.8% compared to those engaged in additional income-generating activities (1.2%). The chi-square p-value was 0.003. Furthermore, households that didn’t rely on saving were more likely to report a substantial threat, accounting for 52.6% compared to those who relied on savings (26.4%).

The cross-tabulation revealed a significant association between residence and perceived financial threat of COVID-19, with a chi-square p-value of 0.000. Urban residents were more likely to perceive COVID-19 as a substantial (44.1%) or moderate (11.2%) financial threat compared to rural residents (17.6% and 6.9%, respectively). In contrast, rural residents were slightly more likely to perceive COVID-19 as “not much of a threat” or “not a threat at all” than urban residents.

Finally, nutritional insecurity was significantly associated with the perceived financial threat of COVID-19, with a chi-square p-value < 0.001. That is households unable to eat healthy or nutritious food are more likely to perceive COVID-19 as a substantial (31.4%) threat compared to households able to eat healthy or nutritious food. This shows the correlation of health, nutrition, and financial stability during crises.

### Brant test of parallel regression assumption

To assess the parallel regression (proportional odds) assumptions for independent variables in the ordinal logistic regression model, the Brant test was conducted. The overall result of the Brant test yielded a chi-square value of 31.017 with 10 degree of freedom and a p-value of 0.0006, indicating a significant violation of the proportional odds assumption for the model as a whole (p-value < 0.05). This result suggests that some of the independent variables violated the proportional odds assumption, and a more flexible model may be required. Among the independent variables included in the regression analysis, the variable gender of the household head, COVID-19 illness worry, engaged in additional income generating activities, and relied on savings violated the proportional odds assumption. On the other hand, the factor’s age of the house hold head, able to buy enough medicine, able to buy enough teff/injera, and employment status satisfied the proportional odds assumption Table 2. As the proportional odds assumption was satisfied partially, the PPOM analysis was conducted using the **gologit2** package in STATA.

**Table 2:** Brant test of parallel regression assumption

Variable	Chi^2^	df	*p* > Chi^2^
Age of HHH	0.912	1	0.339
Gender of HHH	4.375	1	0.036
COVID-19 illness worry	12.953	3	0.004
Able to buy enough medicine	1.069	1	0.301
Able to buy enough teff/injera	3.158	1	0.075
Employment status	2.491	1	0.114
Additional income activities	6.937	1	0.008
Relied on savings	4.183	1	0.041
Overall	31.017	10	0.0006

### The partial proportional odds model: key findings and estimates

The partial proportional odds model was used to determine the determinants of the perceived financial threat of COVID-19, categorized into three levels: “a small threat”, “a moderate threat”, and “a substantial threat”. In this study, the perceived financial threat of COVID-19 was initially categorized into four groups: not a threat at all, not much of a threat, a moderate threat, and a substantial threat. However, because the frequency of the ‘not much of a threat’ category was small and its concept closely related to ‘not a threat at all,’ we decided to combine both ‘not much of a threat’ and ‘not a threat at all,’ renaming the category ‘a small threat’ for the final analysis, which includes three ordinal categories. The PPOM relaxes the proportional odds assumption where necessary, allowing certain variables to have threshold-specific effects. Coefficients are presented as adjusted odd ratios (AOR) with corresponding 95% Confidence Intervals (CI) and p-values. The results are divided into two comparisons: “Substantial threat” versus lower categories, and “Moderate threat” versus lower categories. The interpretation of each key variable is summarized as follows:

For the age of the household head, with each one-year increase in age, the odds of perceiving COVID-19 as a higher financial threat increase by 28%, holding other variables constant [AOR = 1.280, 95% CI: 1.065, 1.538, *p* = 0.008]. The identical odds ratios across thresholds suggest that the proportional odds assumption holds for this variable.

Worry about COVID-19 illness significantly influences perceptions. Those who were very worried about the illness were 54.6% less likely to perceive a substantial financial threat (AOR = 0.546, 95% CI: 0.421–0.709, *p* < 0.001) and 43.8% less likely to perceive a moderate threat (AOR = 0.562, 95% CI: 0.376–0.839, *p* = 0.005) compared to those who are not worried. However, the associations for those who were somewhat or not too worried were not statistically significant. The ability to buy enough medicine was a significant factor, with individuals who can afford medicine being nearly twice as likely to perceive COVID-19 as a financial threat (AOR = 1.901, 95% CI: 1.243–2.907, *p* = 0.003). In contrast, the ability to buy enough teff/injera shows no significant association (AOR = 1.303, 95% CI: 0.964–1.761, *p* = 0.084).

Employment status and additional income activities also significantly influence financial threat perception. Employed individuals are 31% more likely to perceive COVID-19 as a financial threat (AOR = 1.310, 95% CI: 1.056–1.626, *p* = 0.014). Those engaged in additional income activities have much higher odds of perceiving a substantial (AOR = 3.428, 95% CI: 1.624–7.236, *p* = 0.001) or moderate (AOR = 3.043, 95% CI: 1.206–7.677, *p* = 0.018) financial threat. Relied on savings, however, is not significantly associated with perceiving a financial threat, as shown by non-significant p-values in both threat categories (*p* > 0.05). In general, significant factors of financial threat perception include age of the household head, worry about COVID-19, ability to buy medicine, employment status, and engaged in additional income activities. Above all, the gender of the household head, COVID-19 illness worry, engaged in additional income-generating activities, relied on savings violates the proportional odds assumption, which is appropriately addressed using the PPOM. This model effectively handles variables where the proportional odds assumption is violated, producing robust and reliable results for understanding financial threat perceptions during COVID-19 Table 3.

**Table 3:** The multivariate analysis of generalized ordered logit result

Perceived financial threat	AOR	95% CI		*P*-value	AOR	95% CI		*p*-value
Substantial threat					Moderate threat			
**Age of HHH**	1.280	1.065	1.538	0.008	1.280	1.065	1.538	0.008^**^
**Gender of HHH**								
Female	0.860	0.738	1.002	0.053	0.571	0.302	1.079	0.085
**COVID-19 illness worry**								
Very worried	0.546	0.421	0.709	0.000	0.562	0.376	0.839	0.005^**^
Somewhat worried	0.695	0.481	1.005	0.053	0.424	0.169	1.066	0.068
No too worried	0.841	0.631	1.118	0.234	0.883	0.742	1.049	0.159
**Able to buy enough medicine**								
Yes	1.901	1.243	2.907	0.003	1.901	1.243	2.907	0.003^**^
**Able to buy enough teff/injera**								
Yes	1.303	0.964	1.761	0.084	1.303	0.964	1.761	0.084
**Employment status**								
Employed	1.310	1.056	1.626	0.014	1.310	1.056	1.626	0.014^*^
**Additional income activities**								
Yes	3.428	1.624	7.236	0.001	3.043	1.206	7.677	0.018^*^
**Relied on savings**								
Yes	0.792	0.573	1.095	0.158	0.749	0.485	1.158	0.195

## Discussion

This study aimed to assess the perceived financial threat of COVID-19 and its impact on the household’s economic stability in Ethiopia. Significant factors of financial threat perception include the age of the household head, COVID-19 illness worry, the ability to buy medicine, employment status, and engaged in additional income activities. Given the ordered nature of the dependent variable, which is the perceived financial threat of COVID-19, ordinal logistic regression was more appropriate statistical method. However, upon testing, the proportional odds assumption a key requirement for the use of an ordinal logistic regression model was found to be violated. Consequently, the PPOM was performed to determine the significant factors that influence the perceived financial threat of COVID-19. This result is in line with previous studies (Agga and Scott 2015; Guure and Afagbedzi 2022; Liu and Koirala 2012).

The result revealed that roughly 80% of the respondents experienced some level of financial threat of the COVID-19 pandemic, with a majority from these experienced a substantial financial threat. These findings align with other different research outputs (Adamus and Grežo 2021; Goshu et al. 2020; Kuang et al. 2020; Mulugeta et al. 2021). In their findings, the majority of respondents indicated that COVID-19 might pose a substantial threat to their daily lives and economy.

Age of the respondent is a significant factor, where older respondents are more likely to perceive COVID-19 as a higher financial threat as compared to younger. Which is consistent with previous research findings (Nino et al. 2021; Samuel et al. 2022). In their findings, older individuals viewed COVID-19 as a financial threat due to limited income opportunities and increased health expenditure. However, this finding is competing against the findings of other research outputs (Birditt et al. 2021). In their finding, older people reported less pandemic-related stress, less life change, less social isolation, and lower negative relationship quality than younger people. This inconsistency might be occurred due to economic and cultural variations. Older individuals in low income countries may face greater financial stress due to inadequate social safety nets and higher healthcare costs while those in high-income countries might have more stable income. The other reason might be variation in study populations, timing in data collection, and differing methodologies.

The cross-tabulation analyses in Table 1, revealed that the gender of the household head has an association with the perceived financial threat of COVID-19. That is male-headed households reported a higher perception of COVID-19 as a substantial threat (42.0%) compared to female-headed households (19.8%). As the chi-square p-value = 0.017, which is statistically significant, there was an association between the gender of the household head and the perceived financial threat of COVID-19. Even if the partial ordered logistic regression result indicates that the gender of the household head was insignificant, the statistical association in the chi-square result revealed that male-headed households reported a higher perception of COVID-19 as a threat to their livelihood compared to female-headed households. This result is not in line with other studies that showed the disproportionate economic impact of COVID-19 on women, specifically in developing countries (Alsharawy et al. 2021; Chitiga et al. 2021; Hewa-Wellalage et al. 2022). This inconsistency might have occurred because the majority of the households interviewed were male-headed, and COVID-19 threatened all communities regardless of gender. This reasoning is supported by the PPOM analysis, which indicated that although the gender of the household head had a significant relationship with the perceived financial threat of COVID-19 according to chi-square analysis; it was insignificant in the PPOM and violated the proportional odds assumption. Additionally, this contradiction may arise from the fact that most of the interviewed households were male-headed, leading to a dominance of male perceptions in the overall results.

The findings revealed that an individual who can able to buy enough medicine is almost twice as likely to view COVID-19 as a substantial financial threat. This indicates that even if an individual can cover their healthcare costs, they may still perceive COVID-19 as a financial threat due to the pandemic. This finding is consistent with other studies (Billiet et al. 2014; Bruce et al. 2022; Wieser et al. 2020b). Also, employed individuals were more likely to perceive COVID-19 as a financial threat. Which is consistent with other study findings (Wilson et al. 2020). In their findings, job instability due to the COVID-19 pandemic was highly associated with financial threat and economic crises. Similarly, the research findings reported in Ethiopia shows sectors heavily reliant on employment, such as urban services and manufacturing, experienced severe disruptions, heightening financial concerns among workers (Wieser et al. 2020b). While this result might appear counterintuitive, it can be explained by the greater financial obligations and expectations typically faced by employed individuals, such as household expenses and job insecurity during the pandemic.

Those engaged in additional income activities have much higher odds of perceiving COVID-19 as a financial threat. This finding highlights the importance of economic diversification as a coping strategy, particularly in times of financial uncertainty. It aligns with prior studies (Bruce et al. 2022; Hill and Narayan 2020).

### Limitations

This study has several limitations that should be acknowledged. First, the use of a cross-sectional design limits the ability to establish causal relationships between the perceived financial threat and its associated factors. Second, the data were collected through a phone survey, which may introduce selection bias, particularly in rural areas with limited phone access. Third, non-standardized tools were used to assess both the outcome and some independent variables, which might affect measurement accuracy. Lastly, the study relied on self-reported data, which can be subject to recall and social desirability biases. Future studies should consider longitudinal designs and standardized measurement tools to validate and expand on these findings.

## Conclusion

This study aimed to assess the perceived financial threat of COVID-19 and its impact on the household’s economic stability in Ethiopia. The findings revealed that a significant proportion of respondents perceived COVID-19 as a financial threat, which adversely affected their economic stability. Vulnerability to financial threat was notably higher among older individuals, the unemployed, those unable to afford essential medicine, and respondents who expressed heightened concern about illness. In contrast, engagement in additional income-generating activities served as a protective factor. These results underscore the need for policymakers to prioritize inclusive social protection systems, expand access to affordable healthcare, promote employment opportunities, and facilitate income diversification. Such interventions are critical to enhancing household economic resilience and enabling a rapid response to future public health and economic crises. Additionally, future research should consider longitudinal designs to track changes in perceptions over time and incorporate broader economic indicators.

## Data Availability

The data used in this study were obtained from the third round of the COVID-19 High-Frequency Phone Survey of Households (HFPS-HH), conducted by the Central Statistical Agency (CSA) of Ethiopia in collaboration with the World Bank. Access to the dataset was granted by the World Bank upon request. As the data are not publicly available, researchers interested in accessing the dataset should contact the World Bank for permission. Anyone can see the survey brief at: https://www.worldbank.org/en/country/ethiopia/brief/phone-survey-data-monitoring-covid-19-impact-on-firms-and-households-in-ethiopia
